# Parents' knowledge and attitudes toward pediatric concussions in Saudi Arabia

**DOI:** 10.3389/fped.2026.1826731

**Published:** 2026-05-29

**Authors:** Sarah S. Bajuaifer, Ahmed M. Almansour, Bayan Aldawsari, Raghad Almousa, Reema Bin Aqeel, Refa Alghanim, Nouf Almalki, Abdulaziz A. Alkathiry

**Affiliations:** 1 Department of Rehabilitation Sciences, College of Health and Rehabilitation Sciences, Princess Nourah Bint Abdulrahman University, Riyadh, Saudi Arabia; 2 Department of Physical Therapy and Health Rehabilitation, College of Applied Medical Sciences, Majmaah University, Al Majmaah, Saudi Arabia; 3 Health and Basic Sciences Research Center, Majmaah University, Majmaah, Saudi Arabia

**Keywords:** concussion, parental attitudes, parental knowledge, Saudi Arabia, sociodemographic factors

## Abstract

**Background:**

Concussions are considered a public health issue with both short- and long-term physical, cognitive, and emotional consequences. Parents play an important role in recognizing symptoms, seeking care, and managing recovery. In Saudi Arabia, there is limited evidence regarding parental knowledge and attitudes toward pediatric concussions.

**Objective:**

The study assessed parental knowledge and attitudes regarding concussions in Saudi Arabia and examined the influence of sociodemographic factors, such as income, education level, gender, number of children, and history of falls.

**Methods:**

A nationwide cross-sectional survey was carried out across Saudi Arabia from February to April 2025. Parents aged 18 and above with children participated through an online self-administered questionnaire. The survey gathered demographic information, beliefs regarding concussion management, recognition of symptoms, and knowledge and attitudes about concussion.

**Results:**

A total of 524 participants were included in the final analysis. The majority of participants were female (79.2%), Saudi nationals (95.2%), and residents of the central region (68.5%). The age distribution indicated that most participants were between 31 and 50 years old (71.1%). The average score on the Concussion Knowledge Index was 15.4 ± 3.0 out of 25, while the average attitude score was 42.0 ± 6.2 out of 49. Despite this, 63.0% of participants reported not having prior awareness of concussion-related information. CKI scores showed a positive correlation with education level (*ρ* = 0.229, *p* < 0.001) and income (*ρ* = 0.166, *p* < 0.001). There was also a significant positive correlation between knowledge and attitude scores (*ρ* = 0.266, *p* < 0.001). Parents who reported previous awareness of concussions had notably more positive attitudes (*p* = 0.008). No significant differences in knowledge or attitude scores were observed based on gender, geographic region, or history of child falls.

**Conclusion:**

This study provides nationwide cross-sectional survey data on parents' knowledge and attitudes about pediatric concussions in Saudi Arabia. The results highlight the need to develop culturally appropriate educational programs for parents and public health initiatives focused on enhancing early detection and management of pediatric concussions.

## Introduction

Concussion is classified as a form of mild traumatic brain injury (mTBI), and represents a significant global public health concern, particularly among children and adolescents (1). It is the most common type of mTBI, caused by complex physiological responses to biomechanical forces impacting the brain (2, 3). Although often considered temporary, concussions can produce persistent symptoms, which are categorized into four main types: somatic (physical), cognitive, sleep-related, and emotional (4, 5). Symptoms frequently include headaches, dizziness, blurred vision, fatigue, and concentration problems (5). Despite growing awareness, many concussion cases go undiagnosed or unreported (3, 6). Fortunately, most people recover within a few weeks (4). Children are especially vulnerable because of ongoing brain development, active participation in physical activities, and limited ability to recognize or communicate their symptoms, leading to missed diagnoses and delayed treatment (1, 7).

Children's concussions remain a growing public health concern in the United States. By 2021, approximately 3.9% of children aged 0–17 years had been diagnosed at least once with a concussion or brain injury by a healthcare professional. The prevalence of concussions increases with age, with only 0.9% reported among children aged five and under, compared to 8.3% among adolescents aged 12–17. Statistics show that as children get older, they participate in more physical activity and sports and are at a higher risk because of their developmental stage (7).

Concussions are linked to a 40% increased risk of mental health issues in children and adolescents (8). In Saudi Arabia, data on children's traumatic head injuries (THIs) remain limited. A retrospective study by Albargi et al. (2025) of 466 children with concussion found that most were boys (76.6%) over six years old (69.5%), with motor vehicle accidents as the main cause (51.9%). It also found that falls were most frequent in infants (69.8%) (9). Although the causes of concussions vary by age, symptoms often overlap. Adolescents commonly sustain concussions through contact sports, while younger children are more prone to falls and home accidents (10). Regardless of cause, both groups may experience cognitive, emotional, and physical symptoms, such as headaches and behavioral changes (10–12). Parents play a central role in recognizing and managing concussions among children (13). They are typically responsible for identifying symptoms, seeking medical care, supervising recovery, and making decisions regarding returning to school and sports. Previous research has demonstrated that parental knowledge and attitudes toward concussions significantly influence injury reporting, healthcare utilization, adherence to medical recommendations, and recovery outcomes (10, 13). Parents who are more aware of concussion symptoms and risks are more likely to seek timely care and enforce appropriate activity restrictions. In contrast, misconceptions or minimizing attitudes may lead to delayed diagnosis, a premature return to learning and other activities, and prolonged recovery. Despite growing international attention to pediatric concussions, research examining parental knowledge and attitudes toward pediatric concussions in Saudi Arabia remains limited. Existing Saudi studies have generally involved small or specific populations and may not adequately represent the broader community (14, 15). Additionally, little is known about how sociodemographic factors, including income, education level, gender, family size, prior head injury history, and traditional or cultural beliefs, influence parents' understanding and perceptions of concussions. To our knowledge, this study included a larger, more geographically diverse sample than previous Saudi studies on parental concussion knowledge and attitudes. This study aimed to evaluate parents' knowledge and attitudes regarding pediatric concussions in Saudi Arabia, explore how certain sociodemographic factors influence these perspectives, and identify the primary sources parents use to access information about concussions.

## Method

This study was conducted in accordance with the principles outlined in the Declaration of Helsinki. Ethical approval was obtained from the Princess Nourah University Institutional Review Board (IRB: 25-0080). The estimated sample size of 385–400 parents were considered statistically appropriate for this study. It was manually calculated in accordance with data from the Saudi General Authority for Statistics. Approximately 55% of the Saudi Arabian population (around 13 million individuals) is married. Using a 95% confidence level and a 5% margin of error, the required sample size was also calculated using Cochran's formula.

Before enrollment, all participants provided digital informed consent. The study focused on Saudi Arabian parents aged 18 or older who were fluent in Arabic. Those under 18 or without children were excluded. A nationwide cross-sectional survey was conducted between February and April 2025.

Data collection involved a self-administered online questionnaire developed and distributed in Arabic using Microsoft Forms. It was shared through convenience sampling on popular social media platforms in Saudi Arabia, such as WhatsApp and Twitter (X), along with parent networks and community groups. Participants voluntarily completed the survey through an electronic link. The study followed the STROBE guidelines for cross-sectional research (16).

The survey was organized into five sections. Section One obtained informed consent, while Section Two served as a screening tool for exclusion criteria. Section Three collected demographic information and included questions regarding concussion management, as well as traditional and cultural beliefs related to concussion; it also contained two questions on red flags and risk indicators from the Sports Concussion Assessment Tool—5th Edition (SCAT5) (17). Section Four assessed parental knowledge and symptom recognition related to concussions using selected sections (1, 2, and 5) adapted from the Arabic version of the Rosenbaum Concussion Knowledge and Attitudes Survey—Student Version (RoCKAS-ST) (18, 19). The RoCKAS-ST is a 55-item questionnaire consisting of five sections designed to evaluate concussion-related knowledge and attitudes. For this study, only Sections 1, 2, and 5 were used to measure parents' factual knowledge, with minor linguistic modifications to improve clarity for parents. Term such as “athlete” and “player” were replaced with “child” where relevant, keeping the original meaning. These modifications were intended to improve the questionnaire's relevance and comprehensibility for parents. Each correct answer was awarded one point, yielding a total score between 0 and 25, with higher scores indicating greater concussion knowledge. A validity check was performed using three items from the RoCKAS-ST Response Validity Index to ensure response consistency; participants who failed these items were excluded from analysis. Section Five assessed parental attitudes toward pediatric concussions using a seven-item scale adapted from a validated questionnaire by Kay et al. (2017), targeting parents of youth sport athletes (20). Responses were rated on a 7-point Likert scale, resulting in an overall score from 7 to 49. The survey was pretested with 12 parents to ensure clarity and relevance, and language adjustments were made before final distribution.

For descriptive statistics, CKI and CAI results were reported as means and standard deviation. Normality of continuous variables was assessed prior to analysis. Because the data were not normally distributed, nonparametric tests were applied. Spearman's rank-order correlation was used to examine associations between CKI scores, attitude scores, and selected demographic variables. Group comparisons were conducted using the Mann–Whitney *U* test for two-group comparisons and the Kruskal–Wallis test for comparisons involving more than two groups. Statistical analysis was performed using IBM SPSS Statistics for Macintosh (version 28; IBM Corp, Armonk, NY) with the significance level set at *α* = 0.05.

## Results

### Participants' characteristics

A total of 903 participants consented to participate in the study, of whom 326 were excluded based on the survey's exclusion criteria. A total of 577 participants completed the survey. However, 53 participants were excluded after failing the three validity index questions.

A total of 524 participants were included in the final analysis. The majority of participants were female (79.2%), Saudi nationals (95.2%), and lived in the central region (68.5%). Most participants were between 31 and 50 years of age (71.1%). More than half of the participants held a bachelor's degree (57.1%), and the most common monthly income was between 7,001 and 15,000 SAR (42.0%). Nearly half (49.0%) reported that at least one of their children had experienced a head injury-related fall. The demographic characteristics of the participants are summarized in Table 1.

**Table 1:** Demographic characteristics of participants.

Characteristics	*N* (%)
Gender	
Female	415 (79.2)
Male	109 (20.8)
Age group (years)	
18–25	21 (4.0)
26–30	46 (8.8)
31–40	159 (30.3)
41–50	214 (40.8)
51–60	73 (13.9)
> 60	11 (2.1)
Monthly income (SAR)	
None	85 (16.2)
<7,000	86 (16.4)
7,001–15,000	220 (42.0)
15,001–25,000	91 (17.4)
>25,000	42 (8.0)
Nationality	
Saudi	499 (95.2)
Non-Saudi	25 (4.8)
Region of residence	
Central	359 (68.5)
West	53 (10.1)
North	42 (8.0)
East	40 (7.6)
South	30 (5.7)
Education level	
<High school	9 (1.7)
High school	74 (14.1)
Diploma	49 (9.4)
Bachelor's degree	299 (57.1)
Postgraduate	93 (17.7)
Number of children	
One	88 (16.8)
Two	113 (21.6)
Three	97 (18.5)
Four	103 (19.7)
Five or more	123 (23.5)
Number of parents who had a child who experienced a fall involving the head	257 (49.0)

### Concussion knowledge and attitudes

The mean score on the concussion knowledge index (CKI) was 15.4 ± 3.0 out of a possible 25. The mean score on the attitude scale was 42.0 ± 6.2 out of 49. A significant knowledge gap was identified, as most participants (330, 63.0%) reported lacking awareness of concussion- related health information. For those who were aware, general internet sources (e.g., websites) were the most frequently cited source (96, 18.3%), followed by social media platforms (85, 16.2%) and doctors (84, 16.0%). Awareness campaigns and workshops were the least cited sources (27, 5.2%).

Analysis of participants' responses to symptom recognition items revealed notable discrepancies between legitimate concussion symptoms and distractor items. Among the legitimate symptoms, dizziness was the most accurately identified, with 82.6% of participants recognizing it as a post-concussive symptom. Similarly, high recognition was observed for difficulty concentrating (75.6%), headache (74.4%), and difficulty remembering (72.1%). However, other legitimate symptoms were identified with less consistency, such as sensitivity to light (33.5%) and drowsiness (40.6%), indicating gaps in symptom awareness. In contrast, several distractor symptoms that do not typically result from concussions were mistakenly reported at concerning rates. Notably, 64.1% of participants incorrectly reported speech difficulty, and 34.5% reported a reduced breathing rate, indicating significant misconceptions regarding concussion symptoms. Other distractor symptoms, such as arthritis (2.3%), hair loss (1.5%), and weight gain (1.1%), were rarely endorsed, indicating a better recognition of non-concussion-related symptoms. These findings suggested that while the core cognitive and physical symptoms of concussions are broadly recognized, confusion regarding less commonly recognized neurological symptoms persists, and several misconceptions regarding non-concussion symptoms remain prevalent, warranting targeted educational intervention.

### Fall history and management

Nearly half of the participants (257, 49.0%) noted that a child in their care had experienced a fall involving the head. Of these, 76 children (14.5% of the total sample) experienced repeated falls. The most common location for a fall was at home (213, 82.9%). Following a fall, the most common actions taken were visiting the emergency room (35, 13.6%) or taking no action (29, 11.3%). The use of official channels, such as calling 937) which is a 24/7 call center and digital platform providing emergency health services by the Saudi Ministry of Health) (5, 1.9%) or the Sehhaty application, (which is unified national health application provided by the Ministry of Health), (2, 0.8%), was very low.

Out of the 76 participants who reported that their children had experienced repeated head falls, responses to SCAT5-based questions regarding observable concussion signs and red flag symptoms were collected. Regarding observable signs, none of the participants reported witnessing a child lying motionless on the playing surface. Only 3 participants reported observing balance or gait difficulties, such as stumbling or slow, labored movements, while only 1 participant reported signs of disorientation, confusion, or an inability to respond appropriately to questions. In contrast, 10 participants observed a blank or vacant look following the fall, and 19 participants noted a facial injury after head trauma. However, the majority (41 participants) indicated that they had not observed any of the listed symptoms. Regarding red flag symptoms, 5 participants reported neck pain or tenderness, 3 reported double vision, and 3 reported weakness, tingling, or burning sensations in the arms or legs. Severe or increasing headache was reported by 10 participants, while 6 participants noted a seizure or convulsion, and 13 participants reported vomiting following the incident. 8 participants reported loss of consciousness. Notably, no participants reported a worsening of consciousness or increased restlessness, agitation, or combativeness. The majority, 43 participants, however, indicated that they had not observed any of the listed red flag symptoms. Including questions based on SCAT5 observable signs and red flag symptoms was crucial in this study to evaluate the depth of parental knowledge regarding the immediate recognition of concussion-related warning signs following repeated head falls, as timely identification by caregivers plays a critical role in ensuring early intervention, preventing complications, and promoting safer outcomes for children. Of those, only 6 participants (1.1%) believed that traditional treatments, such as massage, might improve symptoms.

### Factors associated with concussion knowledge and attitudes

Spearman's rank-order correlation analysis identified several significant relationships. CKI scores were significantly positively correlated with education level (*ρ* = 0.229, *p* < 0.001) and income (*ρ* = 0.166, *p* < 0.001). Additionally, a significant positive correlation was observed between CKI and attitude scores (*ρ* = 0.266, *p* < 0.001). Normality tests indicated that the data were not normally distributed; thus, nonparametric tests were employed.

The Independent-Samples Mann–Whitney *U* Test and Independent-Samples Kruskal–Wallis Test were used to examine group differences. A statistically significant difference was found in parents who reported being aware of concussions, demonstrating significantly more positive attitudes than those who were unaware (*U* = 27,591.50, *p* = 0.008). No significant differences in CKI or attitude scores were observed based on gender (CKI: *p* = 0.465; Attitude: *p* = 0.053), geographical region (CKI: *p* = 0.389; Attitude: *p* = 0.066), or history of a child's fall (CKI: *p* = 0.254; Attitude: *p* = 0.525). Table 2 provides detailed descriptive statistics for CKI and attitude scores across these variables, while Table 3 details the results of the association tests.

**Table 2:** Descriptive statistics for the concussion knowledge Index and attitude scores across demographic variables.

Variable	Group	*N*	CKI Score (Mean ± SD)	Attitude Score (Mean ± SD)
Overall		524	15.39 ± 3.03	41.97 ± 6.17
Gender	Female	415	15.43 ± 3.06	42.28 ± 5.99
	Male	109	15.24 ± 2.93	40.77 ± 6.69
Region of Residence	Central	359	15.50 ± 3.06	42.37 ± 5.46
	East	40	15.28 ± 2.54	41.98 ± 7.51
	North	42	14.43 ± 3.22	38.86 ± 8.48
	South	30	15.50 ± 2.91	42.83 ± 6.78
	West	53	15.42 ± 3.05	41.21 ± 6.52
Child Experienced a Fall involving the head	Yes	257	15.54 ± 3.09	42.15 ± 5.55
	No	267	15.25 ± 2.97	41.79 ± 6.72
Awareness of Concussions	Yes	194	15.75 ± 3.25	42.20 ± 7.06
	No	330	15.18 ± 2.88	41.83 ± 5.59

CKI, concussion knowledge index; N, Sample size.

**Table 3:** Associations between CKI, attitude, and demographic and participation variables.

Variable	CKI Score		Attitude Score	
	Test statistic	*P*-value	Test statistic	*P*-value
Gender (Male vs. Female)	*U* = 21,593.5	.465	*U* = 19,908.5	.053
Geographic Region	*χ*²(4) = 4.13	.389	*χ*²(4) = 8.80	.066
History of Fall involving the head (Yes vs. No)	*U* = 32,341.0	.254	*U* = 35,406.5	.525
Concussion Awareness (Yes vs. No)	*U* = 28,770.5	.052	*U* = 27,591.5	.008

CKI, concussion knowledge index; U, Mann–Whitney *U* test; χ², Chi-squared test.

## Discussion

This study evaluated parents' knowledge and attitudes regarding pediatric concussions in Saudi Arabia, as parents play an important role in recognizing symptoms after a child's head injury and determining when medical evaluation is warranted. Although the mean CKI score suggested partial concussion-related knowledge, 63.0% of participants reported lacking awareness of concussion-related health information, indicating an important gap in public awareness of concussion-related information. This discrepancy may indicate partial knowledge rather than a complete understanding, as some participants may have answered structured knowledge questions correctly through general reasoning or experience. These findings suggest that the CKI scores should be interpreted cautiously and may not necessarily reflect a strong practical understanding of concussion recognition or management.

Furthermore, parents more frequently recognized symptoms like dizziness, headache, difficulty concentrating, and difficulty remembering. However, they struggled to identify symptoms such as sensitivity to light and drowsiness. This indicates that parents are more familiar with common and basic concussion symptoms but are less aware of subtle or delayed symptoms. This is important because a lack of awareness and understanding of these symptoms could delay monitoring, seeking medical advice, or implementing proper activity restrictions following a child's head injury.

Nearly two-thirds of parents reported lacking awareness of concussion-related health information, highlighting an important gap in public health communication and education. Among parents who reported partial awareness, we found that the internet and social media were the primary sources of information. This indicates that parents usually access concussion-related information using digital sources, friends, or family rather than through professionally guided education. Although these sources provide basic good health information, their content may vary in accuracy and practical usefulness. Therefore, reliance on these sources alone may contribute to persistent misconceptions, particularly if parents do not receive clear guidance on warning signs, symptom monitoring, and when to seek medical care (13, 15, 21). Although education level was significantly associated with CKI scores, the correlation was weak, suggesting that education explains only a small proportion of the variation in concussion-related knowledge. Thus, although higher education and income may be linked to slightly better access to health information or health literacy, these factors do not fully explain parents' concussion knowledge. This finding suggests that concussion-related knowledge gaps persist even among higher-educated and higher-income parents. Therefore, educational interventions should target parents broadly rather than assuming that socioeconomic advantage alone ensures adequate understanding of concussion.

Our results also showed a significant association between parents' awareness of concussions and their attitudes toward concussion safety and management, suggesting that awareness may be associated with stronger perceptions of safety and responses. Similarly, previous research by Altamimi et al. (2022) found that greater parental knowledge was associated with more positive attitudes toward injury prevention at home (15). These findings highlight the important role of education in promoting safer health-related behaviors and reinforce the need for public awareness campaigns.

The high number of female participants in our study should be considered when interpreting the findings. This indicates mothers' involvement in children's daily care and health-related decision-making, which may have increased their likelihood of participating in a survey related to child injury. Although gender was not significantly associated with CKI or attitude scores, female participants may limit the generalizability of the findings to fathers or other male caregivers.

The lack of significant differences by gender, region, and child fall history suggests that knowledge and attitude gaps may be present across different parent groups. Interestingly parents who reported that their child had experienced a fall did not demonstrate significantly higher CKI or attitude scores, suggesting that personal experience with child falls or head injuries may not be sufficient to improve concussion understanding without structured education. Although parents who reported prior awareness of concussions demonstrated significantly more positive attitude scores, the difference was small and should be interpreted cautiously.

Cultural and family factors may also influence how parents respond to child falls and possible concussion symptoms. Some parents may consider minor falls as common childhood events, especially when symptoms are not severe or immediately visible. This may lead families to monitor the child at home, seek informal advice, or delay using official healthcare resources. Therefore, future concussion education should be culturally appropriate and should clearly explain when medical assessment is needed after a head injury. Also, our findings indicate limited parental recognition of observable concussion signs and red flag symptoms following repeated head falls. While some visible signs were identified, important neurological symptoms were less frequently recognized, and many parents reported no observed symptoms. This may reflect difficulty identifying subtle or transient signs. Given the importance of early recognition for timely care, these results highlight the need for targeted education to improve parental awareness of key concussion warning signs.

Additionally, our findings indicate that there is very limited use of official healthcare resources after a fall, including the 937 service and Sehhaty app in Saudi Arabia. This may be because parents are unsure when a fall requires medical attention, do not know about available official services, or rely only on personal judgment unless symptoms appear severe. Providing clearer concussion guidance from official healthcare services, schools, and public health campaigns may help parents recognize warning signs and seek timely advice.

This study has several limitations. Its reliance on self-reported data may introduce recall and reporting bias. In addition, the cross-sectional design prevents it from making causal claims about the relationships among variables. The high proportion of female participants may also limit generalizability to fathers and male caregivers. Although some associations were statistically significant, the observed correlations were weak, indicating that education, income, and CKI scores explained only a small proportion of variation in the related outcomes. Despite these limitations, the findings call for public health initiatives aligned with Saudi Arabia's Vision 2030 goals for a healthier society. Future studies should use longitudinal and qualitative designs to evaluate educational interventions and to better understand cultural, practical, and healthcare-access barriers that influence concussion-safe practices among parents. Future research should also focus on the development and psychometric validation of culturally appropriate instruments specifically designed to assess parental knowledge and attitudes toward pediatric concussion.

## Data Availability

The raw data supporting the conclusions of this article will be made available by the authors, without undue reservation.
